# Psychological Needs and Resources of the Staff in a Pediatric Neurosurgery Ward: A Phenomenological-Hermeneutic Study

**DOI:** 10.3389/fpsyg.2021.751651

**Published:** 2022-01-03

**Authors:** Iacopo Lanini, Debora Tringali, Rosapia Lauro Grotto

**Affiliations:** ^1^Department of Health Sciences, University of Florence, Florence, Italy; ^2^Laboratory for Multidisciplinary Analysis of Relationships in Health Care, UNISER, Pistoia, Italy; ^3^Associazione LAPO O.N.L.U.S., Florence, Italy

**Keywords:** pediatric oncology care, phenomenologic inductive analysis, staff, neurosurgery – pediatric, phenomenologic-hermeneutical research, pediatric brain tumors, qualitative research, pediatric psycho-oncology

## Abstract

Brain tumors are a common form of solid tumors in children and, unfortunately, they are characterized by a very uncertain prognosis. The treatment of this pathology often includes one or more very invasive surgical procedures, quite often in the very first steps of the treatment. Cases of brain tumors in children represent one of the greatest challenges for health care professionals in the domain of pediatric neurosurgery. This is clearly due to the complexity of the therapeutic plan, but also to the nature of the bond that is established between the child, the parents, and the members of the staff during the often-dramatic initial phase of the illness. In this phenomenological-hermeneutic study, we explore both the emotional and organizational needs, as well as the available professional and personal resources of the staff in the Neurosurgery ward of the Meyer Children’s Hospital in Florence (Italy). The ward staff, composed of 7 surgeons, a pediatric neuro-oncologist, 12 nurses, and 4 auxiliary health care professionals, underwent in-depth interviews that were recorded (with the consensus of the participants). The recordings were then transcribed and submitted to content analysis according to COREQ standards. A complex picture of emotional as well as organizational demands emerged from the data. Shared experiences were pointed out, together with more specific and idiosyncratic contents characteristic of different professional roles. The focus of the present paper was twofold, first, we considered the needs that are overtly expressed by the staff, and then we discussed the main sources of their motivational drives. We found that the latter is mainly found in the quality of the therapeutic bond that is established with the children and the family members, together with the deep interest in one’s own professional activity and the effective complementarity and integration of the personal and professional qualities of the staff members within the multidisciplinary caring group.

## Introduction

Tumors of the Central Nervous System are the most common form of solid tumors in childhood, and the second most common form of cancer in the pediatric age (after leukemia), with a mean incidence of 3.3 cases over 100,000 children (Sinzig et al., 2008; Crawford, 2013). Each year, there are around 1,500 new cases of brain tumors in children under 14 years, with a peak of diagnoses in patients between 5 and 10 years. In addition, the concept of degree of malignancy in these cases is sadly peculiar; even the malignant forms that appear to be relatively less aggressive can nonetheless be located in brain areas that are either responsible for the vital life functions or essential for the normal intellectual and psycho-affective development of the child. Thus, we can say that in pediatric brain tumors, the three worst threats for a human being (death, madness, and dementia) apparently co-exist.

The onset of brain tumors can be characterized by a heterogeneous set of clinical conditions, partly correlated with the child’s age at onset (for a review, see Wilne et al., 2007). For instance, in around 50% of the cases starting with an acute episode, the initial symptoms are related to the raise of intracranial pressure, such as headaches, vomiting, and nausea (Jankovic et al., 1998). In other cases, the child is taken to the clinical observation due to very mild symptoms including motor system dysfunctions such as abnormalities of gait and coordination, weight loss, behavioral problems, school difficulties, and sometimes, minimal and transient facial nerves paresis (Wilne et al., 2007). Also, while in adult patients’ symptoms of a focal encephalic lesion are mainly determined by its localization, in the case of children the symptomatology can be aggravated with the presence of general and a-specific behavioral disturbances, found in many different pediatric pathologies.

Finally, it is unfortunately very common for parents of apparently healthy children to find themselves facing a sudden diagnosis of such a severe medical condition that deeply threatens the psycho-physical development of their children, puts their life prospects in doubt, and is almost immediately followed by a long surgical intervention with the doubtful outcome and a real threat of a recovery characterized by a deeply altered emotional state.

On the other hand, there are several therapeutic approaches available to clinicians in these cases, for instance, surgical ablations and similar interventions can be performed (and eventually repeated) to eliminate the damaged tissue or to treat hydrocephalus. However, tissue loss can lead to specific neuropsychological and behavioral deficits. Moreover, it can also have some indirect consequences, such as an overall limitation of the cognitive development of the child. Similarly, radiotherapy, especially at high doses, can compromise the endocrinal equilibrium and is nowadays often contrasted to the option of marrow-ablative chemotherapy with autologous hematopoietic cell transplantation. In any case, long-term survivors must face the prospects of the deep and long-lasting impact of therapies (including side-effects) in many different physical and psychosocial domains, also entailing a significant burden for caregivers and other family members (Gupta and Jalali, 2017). Despite the strain that these therapeutic procedures put on the patients and their families, the prognosis in most cases remains very uncertain – depending on the histochemical characterization of the disease, survival rates 5 years post-onset are reported to be in the range from 60 to 85% (Ward et al., 2014; Gupta and Jalali, 2017).

As already mentioned, these pathologies are extremely heterogeneous (Caciotti et al., 2020). Under these conditions – as one of the pediatricians we interviewed puts it – “*every case is a single case*.” In assuming the care of pediatric patients, who are so complex in many different respects, the health professional staff expose themselves to very strong emotional drifts. Moreover, this often happens in the context of strict contiguity between children, parents, and staff members (Griffiths et al., 2011) and under the pressure of heavy organizational demands. Additionally, the staff is constantly called to carry the heavy burden of responsibility for very difficult decisions.

In this context, the quality of the relational bond established with the parents is a crucial factor in supporting the patients and families in dealing with the challenges, negotiations, and decision-making processes that emerge during the treatments (Pelcovitz et al., 2017; Robinson et al., 2019). A recent systematic review of qualitative data from 33 studies exploring families’ experiences in this context documented how parents feel relieved when health care providers engage in “good” communication and show a genuine effort to provide tailored information and guidance (Young et al., 2021). Similar needs are described in the systematic review completed by Nicklin et al. (2019) regarding adolescent and young adult childhood brain tumor survivors and their caregivers. Authors conclude that “Nuanced communication is needed, not only during treatment but also into survivorship with specific approaches to meet caregiver needs and provide coping skills to manage stressful situations” (ivi, p. 19). Despite this well-documented evidence, at the moment little is known with respect to the way in which healthcare providers do face these requests. According to the single study addressing this issue, parents do not perceive clinicians to be at ease when designing tailored interventions in order to meet family and caregiver needs (Deatrick et al., 2009).

Therefore, in the present study we intend to contribute by exploring the phenomena related to the interpersonal and relational dimensions of caregiving of children with brain tumors, in a phenomenological-hermeneutic perspective (Husserl, 1931; Gadamer, 1960; Vattimo, 1987; Smith, 2003). We consider the texture of human relationships, which instantiate the therapeutic activity of the staff and can either enhance it or make it more difficult (Bleger, 1966, 1967; Carli and Paniccia, 2003; Correale, 2006). We also explore the interplay of the personal dimensions both in the context of the professional bond with the patient and family members as well as within the multidisciplinary professional group (Dixon-Woods et al., 2005; Papini et al., 2011).

Previously, we conducted qualitative research on parents of children suffering from brain tumors, within the same ward (Papini et al., 2011). The study included 16 adults, for the most part, parents of children facing different moments of the course of the disease (diagnostic phase, post-surgical phase, remission, relapse) and in one case, the parents who had lost their child several years before. All participants underwent individual, in-depth interviews (Montesperelli, 1998; Bichi, 2002) that were analyzed following an interpretative phenomenological methodology (Smith, 2003). Results were first shared with the ward staff in a dedicated meeting and later published in the form of extended essays and research reports (Papini et al., 2011; Lauro Grotto et al., 2014).

Each testimony revealed aspects of the tremendous burden which is placed on the families not only by the pathology but by the therapies as well. Some of the accounts depict experiences where increasing parental worries about the child’s symptoms have been met with rejection and disregard by professionals, either family pediatricians or Emergency ward professionals (Papini et al., 2011). On the other hand, all the parents expressed their need to establish an authentic, trustful, and supportive bond with the staff and to be guided through this painful challenge that impacts every domain of their lives. Moreover, within the clinical and professional framework of the ward, they want to be recognized for their difficulties and suffering as human beings. Other critical issues unfolding in parents’ testimonies point out the difficulties in communication and decision making in different phases of the disease, such as diagnosis, relapse, and a transition to a terminal phase, but also in planning the future for their child and the family.

On the other hand, when confronted with these testimonies, numerous members of the medical équipe have confirmed how challenging it was to provide support to the patients and their families. Additionally, some of the staff members have stressed the demand to see their own psychological needs acknowledged and addressed as well. For other clinicians, it was appreciated as a chance to focus on the resources, the rewards, and the *immaterial gifts* that they received from the patients, family members, and colleagues.

Therefore, we decided to develop a qualitative research plan with the aim of exploring experiences of the medical professionals engaged in the cure of children with brain tumors, as well as their families, across different phases of the treatment (diagnosis, active treatment, relapse, transition to terminality, or remission). Upon an explicit request of the director of the Unit and the ward-sister, we decided to extend the study to the entire staff, including the associate sanitary members. The reason for this choice stems from the fact that, in such teams, associate sanitary staff actively support the nurses in managing and care of the practical needs of the young patients’ families and are therefore exposed to the entire emotional burden of the context. For instance, in this ward, they oversee the very subtle task of teaching the parents how to wash the children’s hair, which is not cut anymore after the surgery. After the presentation of the study proposal, all of the staff members accepted to participate in it.

Testimonies provided a very complex and rich picture of demands, institutional and relational issues, concerning the links with the patients and the family members, the équipe dynamics, and the institutional context of the ward; following the suggestions provided by the participants themselves during the feedback meeting, here we devote a specific focus to the analysis of the psychological needs and resources of the professionals in taking care of children suffering from brain tumors.

## Materials and Methods

The research design was developed and subsequently implemented in collaboration with the staff of the Neurosurgery Unit of the Meyer Children’s Hospital in Florence. The study was designed with the aim of acquiring and sharing knowledge about the dynamics that are implied by the experience of caring for children with brain tumors, within a phenomenological-hermeneutic approach [for similar studies in different contexts see Hill et al. (2009) and Robinson et al. (2019)]. It was approved by the Ethical Board of the Department of Psychology (nowadays included in the Department of Health Sciences) of the University of Florence in 2008. Following the results of the first study, involving testimonies of the parents of the children affected by brain cancer, we devised the methodological setting for the present study, which is focused on the experience of medical professionals involved.

The research team, comprising two experts in phenomenological-hermeneutic research (RL and DT) and a psychologist with professional experience in Oncological Intensive Care (IL), presented the aims and methods of the study during one of the weekly group meetings of the Neurosurgery staff, sharing the results of the parents’ interviews with them and analyzing issues that could be explored in the present study. To favor the free recall of experiences and the expression of feeling and perspectives, the testimonies were collected with an open trace (Montesperelli, 1998; Bichi, 2002), that is, based on a list of issues to be addressed with each participant and without any pre-ordered, rigid sequence or standard formulation of the questions. The areas to be explored included the motivations to work in a pediatric neurosurgery ward, first contact with the patient and family members, the communication with patients and families, the emotional burden on the individual and group level, the staff dynamics, significant difficulties, and the major resources when facing a multitude of challenges in taking care of children with brain tumors, the memories of relevant episodes.

The entire staff of the Neurosurgery Unit, including 7 surgeons (all men), a young pediatric neuro-oncologist (man), 12 nurses (1 man and 11 women), and 4 auxiliary health care professionals (all women), took part in the present study, which was completed in 2009. Two of the surgeons had a professional experience of more than 15 years, four had around 10 years of experience and one was still completing his training in pediatric neurosurgery. Among the members of the nursing staff, there were professionals with a long experience in the unit (>5 years), as well as four nurses who had worked in the ward for less than 2 years. Similarly, in the case of the associate sanitary personnel, three of the members had around 2 years of professional experience in the ward while the fourth one was the most experienced, having worked in the Neurosurgery unit for more than 15 years at the time of the study.

In-depth interviews were conducted inside the Hospital but not inside the ward itself. Every interviewee conducted from two to four interviews per week and each interview was performed in a single session, whereas the duration of the interview spanned from 50 to 80 min. Systematic debriefing sessions were found to be necessary to distribute the emotional burden within the research group. The interviews were recorded and subsequently transcribed, with the consensus of each participant. Afterward, interviews were discussed across several meetings of the researchers’ group, where every meeting was dedicated to the familiarization with two to three interviews at a time. During this preliminary phase of the analysis, researchers focused on familiarizing themselves with the material and the analysis of their own emotional responses during the interviews.

In the next phase, using the Interpretative Phenomenological Analysis (Mantovani and Spagnolli, 2003; Smith, 2003; Reid et al., 2005) the material was reorganized into general thematic areas, and subsequently into phenomenological categories within each of them. The phenomenological analysis was performed based on the methodological setting used during the interviews, by extracting and categorizing units of text from the testimonies. This was performed individually by all researchers and various iterations of the process were found to be necessary to reach a consensus in the researchers’ group.

Once the phenomenological categories were denominated, the group produced the selection of the compelling examples from the participants’ quotes for each one, according to the standards defined by the COREQ (Consolidated Criteria for Reporting Qualitative Research) checklist (Tong et al., 2007). The process was based on the analysis of both the shared and the divergent contents. Results were presented to the participants in a feedback group meeting to discuss and validate interpretations established by the researchers’ group (Tong et al., 2007). The focus of the present paper is on two general themes that were found to be of particular interest to the staff during the feedback meetings: the psychological needs expressed in the testimonies and the resources and motivational drives of the participants.

## Results

A complex picture of personal, professional as well as organizational, and institutional demands emerge from the data. Here, we will focus on themes concerning needs that are overtly expressed by the staff and on the sources of their motivational drives. Emergent themes derived from the testimonies are described in Table 1.

**TABLE 1:** A synopsis of the phenomenological categories derived from the testimonies, related to the two thematic domains of the psychological needs and resources of the staff.

The psychological needs expressed by the participants	The resources and motivational drives experienced by the participants
Need to receive psychological support for the staff and for the patients.	Acknowledgment and safeguard mechanisms within the équipe dynamics.
Need for frankness.	The chance to learn from experience.
Need for emotional boundaries.	The “fundamental childhood.”
Need for clinical and ethical boundaries.	Knowing that the bond made was not vain.
Need to leave room for hope.	
Need to express sadness.	

### The Psychological Needs Expressed by the Participants

#### Need to Receive Psychological Support for the Staff and the Patients

In general, the staff expressed high levels of personal suffering related to the kind of experiences that are faced at work. A nurse said:

*Here you work just in the present, there is no future here… I think you can work here 2, 3 at most 4 years, then you must go… me too, I think I will leave in about a couple of years… Obviously, elderly died too, but with older people it was different… I mean, with kids, it is just not on… (nurse 4).*

Lack of support may entail personal difficulties, as expressed by a surgeon:

*“Having somebody with whom I can get things off my chest when I need it… it is not that I don’t feel upset when I diagnose a brain tumor and I have to tell the family… it is not that I don’t feel anything, I feel bloody upset! And then it ends up that I have to act as if I am the lucky one, then I have to, let’s say, kind of forget what’s happened… and somehow try to absorb what’s happened on my own… so I can get tense, I get home and my face is drawn and haggard…” (surgeon 4).*

Lack of continuity and support is perceived as a long-term threat to therapeutic achievements. A pediatrician recalls an episode in which he had a conversation with the mother of an adolescent patient, who was back at home far away from Florence after the treatment. Particularly, he was worried about the lack of continuous professional support for the young boy:

*“I said: ‘Look, we need to look for someone for the boy, not only his friends, because of course, it is good to be with friends and socialize… but he needs someone to give him support because he is not like other boys’ (…) A 14-year-old kid who has death thoughts, is it normal? (…) I can’t stand it…We did so much to get you over the illness and now, are you kidding?” (neuro-oncologist).*

#### Need for Frankness

Surgeons report the need to institute overt communication with the family and the affected child. A surgeon stresses this issue:

*“Frankness, absolute frankness, with the parents and the kid, frankness, even with the kid… yes! Yes! Yes, I want the kid to be present during the dialogue with the parents (…) because if he does not know what we are talking about, he obviously thinks the worst (…) the child has to know what’s the matter with him, what he has to face…” (surgeon 6).*

Nevertheless, frankness has to be balanced by careful consideration of the patient’s and the family’s conditions. A pediatrician shares his point of view with us:

*As a pediatrician… it is not possible to be totally frank. To be honest, even with the older ones, I have never been able to tell them directly ‘look this is a severe illness and you aren’t going to make it’… And he goes back to his experience with the parents: “But even to be as straightforward as I am with the parents, that is to say ‘*look that’s how it is*’, because parents do not understand… the problem is, they just don’t understand. In other words, you can tell them ‘*look the illness is progressing, time’s getting short, we have to try to make him feel as well as possible*’, they just don’t understand, they keep on asking you: so what shall we do now? So, what can we do?” (pediatrician).*

#### Need for Emotional Boundaries

Personnel reports the need to keep a certain level of control over the degree of emotional involvement with patients and families. This is more evident in the case of the surgeons and they clearly state this:

*“Getting involved? Never! Families expect everything except that you get involved… you can’t treat patients if you are close!” (surgeon 6). “…Because it’s no use for me and it’s no use for them, because I need to be cool enough to face anything whatsoever” (surgeon 1).*

But even nurses who are used to greater intimacy with patients report experiences that are so intense as to be felt like a kind of intrusion. Consider the following testimony:

*It has often happened to me that I go home and I have a kid on my mind… this has recently upset me quite a bit… often there is a little boy or girl that, as we say, that I cannot stop thinking about… because I go home and I have that kid on my mind. Then the worst case was the one of a little girl, that they took away… she had three quarters of her cerebrum … all tumor! So then she died, and this has left me a bit… because I was not able to put up a barrier; I clearly remember that I was in the gym and this little girl came to my mind, just like that, suddenly. And then you feel bad, because you get attached to… Yes I really felt it, I was already thinking of her, but this fact that I was there and suddenly she came to my… This really hurt me. (nurse 9).*

Sometimes, the feelings of threat that spill over the spatio-temporal boundaries between the work and life originate from the comparisons with one’s own family members. A nurse said:

*Especially when you have kids at home… you tend to make the comparison… fears are taken home. (nurse 4).*

#### Need for Clinical and Ethical Boundaries

The issue of boundaries comes back in clinical practice as well. Particularly, in the form of the questions regarding the effective possibilities of healing, and ones about the limit after which lives lose their meaning. Staff members report their continuative efforts aimed at avoiding useless therapies and preserving the patients’ quality of life:

*“We must absolutely try to heal, but heal with an acceptable quality of life, this is essential… in my opinion it is absolutely illogical that we give a 5-year-old kid 6 more months with suffering… we must try to heal; if we manage that’s good, if we don’t the kid dies. That’s an end to it. And this is essential…” (surgeon 6).*

Sometimes clinical issues are perceived in the form of a moral dilemma as well:

*What should we guarantee, should we guarantee quality of life for a few years with the risk that it (the tumor) can reappear and that it must be removed again, and it may be inoperable, or should we free him from the disease and sentence him to a wheelchair? (surgeon 1).*

#### Need to Leave Room for Hope

Regardless of the situation, all staff members highlight the need to leave room for hope. A surgeon explains his attitude in the communication with the families:

*As far as I am concerned, I try to tackle the issue with parents by explaining the problem, always leaving a window open as far as it’s possible… I leave a window open even in the most difficult cases, the ones that appear to be most complex to treat, more difficult to resolve completely, I leave room for hope (surgeon 2).*

And pediatrician adds:

*We must always leave room for hope, always! Even the youngest ones realize, even kids as young as 3 or 4, they realize perfectly they are about to die, but hope, you must manage to communicate with your eyes, with small gestures that they can still make it; clearly, how could you do this with words… (neuro-oncologist).*

Some members of the staff explain that hope is necessary to them in order to protect their own functioning at work. One of the associate sanitary members clearly states that

*There must be hope for them, but also for us because otherwise what we do is useless! (A.H.W. 1).*

Here the ward-sister refers to what she is used to reminding her colleagues:

*A wake is for the dead, and as long as a child is alive, I don’t want you standing round the bed looking at him, saying ‘poor thing, poor thing…’ we’ll say that when he is dead! While he is alive, he is alive!” (ward-sister).*

#### Need to Express Sadness

Despite the effort to escape from them, feelings of profound sadness and sorrow are always present as the dark side of hope. These may emerge from the very beginning when receiving a patient in the ward for the first time:

*At morning, when they enter the Hospital, they are received in the Ward, we explain everything to them, how things work here, how does the Ward work… blood test and… placement in bed… because in fact, we place them in bed… [she sighs] (nurse 1).*

Or they can be, in some way, postponed until the moment when turning away from them becomes impossible:

*This thing of the relapse, sometimes I say… you see these creatures in agony, because we had a kid that was at his thirteenth surgery… I mean, you don’t know… It makes you feel sad, sadness, that’s all! You ask to yourself why! Nothing else… these children comes at hand, that are children… defenseless, they are suffering a lot… it makes you feel sad, that’s it, it is the only thing that comes to my mind in those moments, because there isn’t a lot you can do for them… the only thing you can plan to obtain is that they do not have pain… (nurse 5).*

### The Resources and Motivational Drives Experienced by the Participants

#### Acknowledgments and Safeguard Mechanisms Within the Équipe Dynamics

In general, staff members appear to be well-aware of the influence of organizational factors in preserving both their professional and personal wellbeing. They also highlight the existence of different “relationship styles,” characteristic of specific institutional settings. A nurse said:

*Well, first of all, when you go to a new ward you first look to see how the work is organized, what your colleagues are like, if they get on well together, (…) and this is already positive, because I see I am in a ward where people try to help each other… namely if you are having problems with a child ‘Look, don’t worry, I will do it…’ well, this is really important. Secondly, that things are on the level, no back-stabbing… and, for the moment, that’s what it’s like here in neurosurgery! (nurse 6).*

The acknowledgment of the contributions of the different figures is also a factor that promotes the perception of cohesion and job fulfillment:

*On this ward, unlike others where I’ve been, doctors listen to nurses, and that is something that gives you satisfaction, because we are the ones who are always in touch with the kids… (nurse 5).*

At a more personal level, all staff members refer that the acknowledgment of their efforts and the gratitude of the patients’ relatives are a source of huge satisfaction. One of the associate sanitary personnel underlines the importance of being recognized over and above her professional role:

*Even the fact that they know your name when they come and see you, even the fact that they bring you a little present, you know it has a symbolic meaning, but even this fact that they know you by name when they come and say hello, even this gives you satisfaction… (A.H.W. 3).*

In this ward, tough and painful events take place and the occasions for sharing emotions are precious. A nurse explained:

*When there is time, we gather together; during the night shift we exchange confidences. Some colleagues told me about the young children who were in the Ward and died. Sometimes the doctors stay with us as well… (nurse 3).*

And another nurse added:

*You share it, within some limits, the limits connected with the ones who are around you. These are Wards in which, in the end, there are heavy situations where you more deeply know your colleagues with respect to other situations; therefore you know – more or less – the way in which they would react, you know the ones you can share with and the ones you have to support… (nurse 4).*

As a final contribution to this theme, we would like to go back to a particular passage of the testimony of the ward-sister, where she explicitly illustrates the way in which the different reactions and attitudes of the staff members all find their own space, even more, how they sum up in the therapeutic action of the group with respect to the child:

*In reality, there are two or three factions, if we want to call them like this, because here are those with experience, they have the weight of experience on their shoulders and they are also better at managing certain kinds of situations, either because they have lived them more intensely because they come from Intensive Care on Neonatal Intensive Care, they have seen so much and by now they know how and what to do. Then there is the newcomer who arrives, who perhaps arrived yesterday, and finds a child who is very ill and she breaks down, because straightaway she identifies with the situation ‘my God, what can we do, we can’t go on like this, if I see a child as ill as this I can’t bear it’… but actually this stage passes too. Then there is the majority of the group who has been with us more or less since Dr G. arrived, that they have these moments of wavering in old cases, but with new cases they find it easier to relate to them… In the end, by and large, the way staff behaves towards a child who is ill is more or less the same in all situations. (ward sister).*

#### The Chance to Learn From Experience

At a more personal level, “knowing how to be open” to each other entails the chance to turn experience into an opportunity for personal and existential growth. As one of the nurses tells us:

*“You know who to confide in or who needs cheering up rather than somebody else, because you know how they’ve taken it and this is crucial, because if you do not know the people you’re working with, you can’t be open with them, and these are wards where you can expect a lot from people, as to make them think, to reason, to consider facts of life that later have an effect on your own life as well.” (nurse 9).*

However, it seems that it is in the relationship with the children that the most meaningful and valuable learning experiences take place. During one of the accounts, a nurse pointed out the crucial role of the contact with the young patients for her motivational drive:

*It is much easier with children; a child, when he has no pain, he is happy… and then it depends on… there are the older kids that… maybe when they are 14, mentally they are already 30… meaning that they have this delicate way of behaving to their mum or their parents; sometimes I have seen a kid reassuring his mum while going to the operating theatre… Well, these are things that fill you with emotion, beautiful things that not everybody has the chance to feel, because in this job you meet certain realities that are quite unique, things that for society are left in the dark, you don’t find out about them; and I think that this is the main reason… (nurse 7).*

#### The “Fundamental Childhood”

Furthermore, some operators underline the importance of being in touch with what one of them calls *the fundamental childhood*. Talking about children he says:

*They usually give you this feeling of life, it’s there, it’s strong, despite the context of the illness. (nurse 9).*

The deep emotional bond that arises in the contact with the child, with its enthusiasm, shyness, attachment, and basic trust, is felt like a kind of vital source for the staff members.

*You get such emotions, priceless emotions, you could not buy them for… really… because you know that there is a kid that is out of Florence and tells his mum: Ah, I want to go back to see Lapo, there is a kind of family tie that goes beyond, way beyond the doctor-patient relationship, way beyond… (oncologist).*

An emergent feature of this vitality is that it is perceived as long-lasting, something that persists along the time:

*With kids it stays with you… the memories, even the silly things you said, or the laughs you had or when you cried anyway. (A.H.W. 1).*

#### Knowing That the Bond Made Was Not Vain

And in fact, operators in this ward devote great care to the material and immaterial signs of their bond to the children and their families. A nurse reminds:

*They thanked us so much in fact; then I went on holiday, and they left me a written message, when I came back I found this card, written by Francesca’s parents… well, there is nothing special to say but… I can remember this, and how it made me feel, intensely… then I saw them again during a check-up, after a month they came for a check-up, and they greeted me with great affection, in fact… (nurse 7).*

Binding to someone is making an investment. Here an auxiliary health professional shares with us her way of keeping for her the awareness that not everything comes to an end with separation:

*I say to myself, well don’t think about it now, because there are children who died and this feeling was a bit more… But life goes on, that child always stays in me, the thought of that child… but then, OK, in any case I have to carry on working, but then this thing, I can stand it… a part of me, well, I say, a part of me… he has had it. (A.H.W. 2).*

## Discussion

Severe illness is an emotionally dense experience at any age. However, when it takes place in the developmental age, the psychological holding system that allows for its elaboration cannot be considered as confined to the patient’s mind (Oppenheim, 2004). It is the relationship with the family members that play a crucial role in making these experiences bearable for the child (Kazak, 1989; Kazak et al., 1994; Pelcovitz et al., 1996; Meitar, 2004; Cerit et al., 2017; Lopez et al., 2019). The fact that the quality of this relationship has a significant influence on a child’s long-term adaptation and the psychological outcome of the treatment does not come as a surprise (Gupta and Jalali, 2017; Nicklin et al., 2019). Due to the nature of the therapeutic bond, the medical staff is directly included in the holding process (Zonza, 2012; Fioretti et al., 2014). It is thus crucial to observe the extent to which caregivers (both medical professionals and family members) are capable of facing such a threatening experience. The ability to perform and cope in such an emotionally demanding scenario is in turn determined by the feeling of being understood and supported as well as by seeing their own needs and difficulties acknowledged, both in the present – within the holding staff dynamics – and in the prior personal and professional experiences (Meadors and Lamson, 2008).

In the present study, we have conducted in-depth interviews with the members of one of Italy’s foremost medical teams treating children affected by brain tumors. The analysis of their testimonies has revealed the pivotal role of the integration of the various personal attitudes and disciplinary perspectives for the effective *holding* of the intense emotional burden inherent in this type of cure. Particularly, while the surgeons and the doctors have to trace the path and oversee the evolution of the therapeutic process, they are also called to help create an adequate and supportive context for the parental decision-making processes. Furthermore, the nurses represent an essential part of the communication network involving the medical personnel, patients, and respective families, in which it is often said that they are “the doctors’ eyes and the parents’ voice,” and their mediation plays an important role in the physical and psychological wellbeing of the child and the parents. Just as importantly, we would like to mention the contribution of the associate sanitary personnel, who represent a key part in providing the day-by-day support to the families and whose presence is vital for carrying the emotional burden of these experiences, together with the medical staff (“*Here, all of us, we are a single équipe*,” said one of the surgeons during a preliminary meeting with the researchers).

Overall, the study took place in an atmosphere of strong emotional commitment of everyone involved. Not a single member of the ward refused to participate in the study. On the contrary, some of the participants even underlined that it was the first time that they were somewhat formally asked to express and share their feelings, emotions, and points of view, and none of them wanted to miss the opportunity to do so. Indeed, our study documented how the sharing of the daily experiences and the emotional burden took place in the group. During late-night meetings, the events of the day were commented on and compared with the individual memories and grieves as well as the history of the ward, thus creating kind of a *delocalized and informal holding ambiance*.

Taking care of the children affected by brain tumors represents an immensely demanding challenge, marked by high levels of uncertainty and a wearing sequence of high and low points during the course of the disease (improvements, complications, etc.). Both the objective and subjective factors weigh in on the emotional load on the personnel. For instance, sometimes the prevalent need within the group seems to be a reestablishing of the real constraints, such as the spatio-temporal boundaries of the work. Other times the more subjective factors are seemingly prevailing, such as an elusive emerging emotion that has to find its way to be expressed and shared or a particular defensive reaction that has to be perceived as possible and granted within the caring group.

The present study highlights the centrality of the relational dimension in the therapeutic acts and life experiences of the staff members, in this dense and intricate context. Each participant approached this complex emotional reality differently. These particular processes of establishing, losing, and re-establishing contact can be felt at various levels (Bleger, 1966): as embodied by different people, as personified through the distinct roles, but even as belonging to the inner world of each one. Thereby, each one is represented and integrated into the therapeutic group over and over again. Being capable of remaining available to a truthful and respectful interpersonal exchange, within one’s limits and sometimes despite them, accepting painful turns and experiencing feelings of fear, anger, uncertainty, and doubt seem fundamental in the process of personal growth and development of a richer professional competence for each member of the group.

To our knowledge, this is the first study based on first-hand accounts of healthcare professionals taking care of children with brain tumors in a neurosurgery unit. Nonetheless, some of the themes emerging from our data can be fruitfully confronted with results coming from different pediatric oncology settings (Zander et al., 2010; Taylor and Aldridge, 2017; Rocque et al., 2018; Slater et al., 2018; Nicklin et al., 2021). Morris and Morris (2017) undertook a qualitative study with the aim of exploring the experiences of the medical staff in a very stressful pediatric setting, characterized by high staff burnout, anxiety, and high turnover rates, namely in a pediatric bone marrow transplant unit. Participants’ reports unveiled the importance of numerous factors for the overcoming of the major relational, personal and professional challenges posed by this type of work. Among others, a strong commitment to providing trustworthy and respectful care and the opportunity to share one’s experiences with others, instead of facing them on one’s own. Similarly, in a recent assessment of a staff wellbeing program introduced at Queensland Children’s Hospital, members highlighted, among other aspects, “a positive effect on awareness of self-care, addressing risks to resilience, seeking support from trusted colleagues, coping with critical incidents, and the ability to interact positively with patients and families” (ivi, p. 67). Furthermore, the analysis of the participants’ suggestions indicated a general appreciation of *individual* abilities’ training programs (such as mindfulness and other self-care abilities) but it also revealed a pressing need to address the work’s challenges in the context of *group* activities [such as formal and informal debriefing sessions, team care activities and regular supervisions (ivi, see Table 5)].

Finally, the themes that emerged from our study show certain similarities with the results of a recent qualitative exploration of staff motivations and resources in a pediatric palliative care setting (Vargas et al., 2016). In both instances, the teamwork and the opportunity to express and share emotions within the multidisciplinary équipe are regarded as protective factors (but only if the attention to the group dynamics and the creation of a good working environment are recognized as important targets of intervention for the Hospital management). Other protective factors include recognizing one’s limits and nevertheless making oneself useful for the patients and their family members and the chance to “connect more and establish closer relations, leaving a lasting impression” (ivi, see the “Working with patients and families” thematic domain). Given this pattern of similarities, future studies could explore if potentially similar transformative processes (the metamorphose, in the authors’ terms) occur among the pediatric neurosurgery personnel in the course of their professional growth.

### Clinical Implications

The results of the present study extend the acknowledgment of the role of the relational, interpersonal, and group factors for the quality of medical care in the neurosurgery setting of children suffering from brain tumors. We highlight the importance and the value of the institutional focus on relational and communication factors for the improvement of the standards of care for both patients and their families in the context of the pediatric neurosurgery ward. Finally, we stress the need for careful consideration of the issues pertinent to the well-being, the personal and professional growth of the staff members.

## Data Availability Statement

The datasets presented in this article are not readily available because the entire records and transcriptions of the interviews are identifiable data, only anonimized partial materials can be shared. Requests to access the datasets should be directed to RL, rosapia.laurogrotto@unifi.it.

## Ethics Statement

The studies involving human participants were reviewed and approved by the Ethical Board of the Department of Psychology (nowadays included in the Department of Health Sciences) at Florence University. The ethics committee waived the requirement of written informed consent for participation.

## Author Contributions

All authors listed have made a substantial, direct, and intellectual contribution to the work, and approved it for publication.

## Conflict of Interest

The authors declare that the research was conducted in the absence of any commercial or financial relationships that could be construed as a potential conflict of interest.

## Publisher’s Note

All claims expressed in this article are solely those of the authors and do not necessarily represent those of their affiliated organizations, or those of the publisher, the editors and the reviewers. Any product that may be evaluated in this article, or claim that may be made by its manufacturer, is not guaranteed or endorsed by the publisher.

## References

[B1] Bichi R. (2002). *L’intervista Biografica.* Milano: Vita e Pensiero Università.

[B2] Bleger J. (1966). *Psicoigiene y Psicologia Istitutional.* Beunos Aires: Paidós.

[B3] Bleger J. (1967). *Simbiosis y Ambigüedad, Estudio Psicoanalíco.* Beunos Aires: Paidós.

[B4] Caciotti C. Fleming A. Ramaswamy V. (2020). Advances in the molecular classification of pediatric brain tumors: a guide to the galaxy. *J. Pathol.* 251 249–261. 10.1002/path.5457 32391583

[B5] Carli R. Paniccia M. R. (2003). *Analisi Della Domanda.* Bologna: Il Mulino.

[B6] Cerit K. K. Cerit C. Nart O. Eker N. Kiyan G. Dagli T. (2017). Post Traumatric stress disorded in mothers of children who have undergone cancer surgery. *Pediatr. Int.* 59 996–1001. 10.1111/ped.13343 28613013

[B7] Correale A. (2006). *Area Traumatica e Campo Istituzionale.* Roma: Edizioni Borla.

[B8] Crawford J. (2013). Childwood brain tumors. *Pediatr. Rev.* 34 63–78. 10.1542/pir.34-2-63 23378614

[B9] Deatrick J. A. Mullaney E. K. Mooney-Doyle K. (2009). Exploring family management of childhood brain tumor survivors. *J. Pediatr. Oncol. Nurs.* 26 303–311. 10.1177/1043454209343210 19837960 PMC2846323

[B10] Dixon-Woods M. Young B. Heney D. (2005). *Rethinking Experiences of Childhood Cancer: a Multidisciplinary Approach to Chronic Childhood Illness.* London: Open University Press.

[B11] Fioretti C. Lauro Grotto R. Tringali D. Padilla Muñoz E. M. Papini M. (2014). Caring for children with brain tumors in an oncology ward: a phenomenologic-hermeneutic study. *J. Pediatr. Neonatal Individualized Med.* 3 1–12.

[B12] Gadamer H. G. (1960). *Wahrheit und Methode.* Tuebingen: Mohr.

[B13] Griffiths M. Schweitzer R. Yates P. (2011). Childhood experiences of cancer: an interpretative phenomenological analysis approach. *J. Pediatr. Oncol. Nurs.* 28 83–92. 10.1177/1043454210377902 20739585

[B14] Gupta P. Jalali R. (2017). Long-term survivors of childhood brain tumors: impact on general health and quality of life. *Curr. Neurol. Neurosci. Rep.* 17:99. 10.1007/s11910-017-0808-0 29119343

[B15] Hill K. Higgins A. Dempster M. McCarthy A. (2009). Fathers’ view and understanding of their roles in families with a child with acute lymphoblastic laukemia: an interpretative phanomenologic analysis. *J. Health Psychol.* 14 1268–1280. 10.1177/1359105309342291 19858345

[B16] Husserl E. (1931). *Die Krisis der Europaeishen Wissenschaften und der Transzendentale Phaenomenologie.* Nijhorff: Den Haag.

[B17] Jankovic M. Conter V. Ferrari E. Colombini A. (1998). “Diffusione e classificazione dei tumori infantili,” in *Tutti Bravi. Psicologia e Clinica del Bambino Malato di Tumore*, ed. Saccomanni R. (Milano: Raffaello Cortina).

[B18] Kazak A. (1989). Families of chronically ill children: a systems and social-ecological model of adaptation and challenge. *J. Consult. Clin. Psychol.* 57 25–30. 10.1037/0022-006x.57.1.25 2647800

[B19] Kazak A. Christakis D. Alderfer M. Coiro M. J. (1994). Young adolescents cancer survivors and their parents: adjustment, learning problems and gender. *J. Fam. Psychol.* 8 74–84. 10.1037/0893-3200.8.1.74

[B20] Lauro Grotto R. Tringali D. Papini M. (2014). *I Tumori Cerebrali Infantili: Relazioni Di cura.* Bologna: Maggioli Editore.

[B21] Lopez R. Glaser A. Kwok-Williams M. Boele F. (2019). Long-term issues and supportive care needs of adolescent and young adult childhood brain tumour survivors and their caregivers: a systematic review. *Psychooncology* 28 477–487. 10.1002/pon.4989 30657618

[B22] Mantovani G. Spagnolli A. (2003). *Metodi Qualitativi in Psicologia.* Bologna. Il Mulino.

[B23] Meadors P. Lamson A. (2008). Compassion fatigue and secondary traumatization: provider self care on intensive care units for children. *J. Pediatr. Health Care* 22 24–34. 10.1016/j.pedhc.2007.01.006 18174086

[B24] Meitar D. (2004). “The family of the child with cancer,” in *Psychosocial Aspects of Pediatric Oncology*, eds Kreitler S. Ben Arush M. V. (Hoboken, NJ: Wiley), 389–414.

[B25] Montesperelli P. (1998). *L’intervista Ermeneutica.* Milano: Franco Angeli.

[B26] Morris C. F. Morris E. J. (2017). The practices and meanings of care for nurses working on a pediatric bone marrow transplant unit. *J. Pediatr. Oncol. Nurs.* 34 214–221. 10.1177/1043454216688637 28415959

[B27] Nicklin E. Pointon L. Glaser A. Sarwar N. Kwok-Williams M. Debono M. (2021). Unmet support needs in teenage and young adult childhood brain tumor survivors and their caregivers: “It’s all the aftermath, and then you’re forgotten about”. *Supportive Care Cancer* 29 6315–6324. 10.1007/s00520-021-06193-x 33861364 PMC8464553

[B28] Nicklin E. Velikova G. Hulme C. Rodriguez Lopez R. Glaser A. Kwok-Williams M. (2019). Long-term issues and supportive care needs of adolescent and young adult childhood brain tumour survivors and their caregivers: a systematic review. *Psychooncology* 28 477–487. 30657618 10.1002/pon.4989

[B29] Oppenheim D. (2004). “The child’s subjective experience of cancer,” in *Psychosocial Aspects of Pediatric Oncology*, eds Kreitler S. Ben Arush M. V. (Hoboken, NJ: Wiley), 111–138.

[B30] Papini M. Tringali D. Lauro Grotto R. (2011). *La Nostra era Una vita Normale.* Savona: Sorbello Editore.

[B31] Pelcovitz D. Goldenberg B. Kaplan S. Weinblatt M. Mandel F. Meyers B. (1996). Posttraumatic stress disorder in mothers of pediatric cancer survivors. *Psychosomatics* 37, 116–126. 10.1016/S0033-3182(96)71577-3 8742539

[B32] Pelcovitz D. Goldenberg B. Kaplan S. Weinblatt M. Mandel F. Meyers B. (2017). Post-traumatic stress disorder in mother of pediatric cancer survivors. *Psychosomatic* 37 116–126. 10.1016/S0033-3182(96)71577-3 8742539

[B33] Reid K. Klowers P. Larkin M. (2005). Exploring lived experience. *Psychol.* 18, 18–23.

[B34] Robinson J. E. Huskey D. Schwartz J. Weaver M. S. (2019). The many roles of the rock: a qualitative inquiry into the roles and responsibilities of fathers of children with brain tumors. *J. Pediatr. Oncol. Nurs.* 11:113. 10.3390/children6100113 31614522 PMC6826713

[B35] Rocque B. G. Cutillo A. Zimmerman K. Arynchyna A. Davies S. Landier W. (2018). Distress and psychosocial risk in families with newly diagnosed pediatric brain tumors. *J. Neurosurg. Pediatr.* 23 40–47. 10.3171/2018.7.peds18297 30485209 PMC6944277

[B36] Sinzig M. Gasser J. Jauk B. Hausegger K. A. (2008). Brain tumors in childwood. *Der Radiologe* 48 439–446. 10.1007/s00117-008-1649-2 18493733

[B37] Slater P. J. Edwards R. M. Badat A. A. (2018). Evaluation of a staff well-being program in a pediatric oncology, hematology, and palliative care services group. *J. Healthc. Leadership* 10 67–85. 10.2147/jhl.s176848 30532609 PMC6241860

[B38] Smith J. A. (2003). Beyond the divide between cognition and discourse: Using interpretative phenomenological analysis in health psychology. *Psychol. Health* 11 261–271. 10.1080/08870449608400256

[B39] Taylor J. Aldridge J. (2017). Exploring the rewards and challenges of paediatric palliative care work – a qualitative study of a multi-disciplinary children’s hospice care team. *BMC Palliat. Care* 16:73. 10.1186/s12904-017-0254-4 29246136 PMC5732535

[B40] Tong A. Sainsbury P. Craig J. (2007). Consolidated criteria for reporting qualitative research (COREQ): a 32-item checklist for interviews and focus groups. *Int. J. Qual. Health Care* 19 349–357. 10.1093/intqhc/mzm042 17872937

[B41] Vargas M. R. Mahtani_Chugani V. Solano Pallero M. Rivero Jiménez B. Cabo Dominguez R. Robles Alonso V. (2016). The transformation process for palliative care professionals: the metamorphosis, a qualitative research study. *Palliat. Med.* 30 161–170. 10.1177/0269216315583434 25895537

[B42] Vattimo G. (1987). Ermeneutica come nuova koinè. *Aut-aut* 3 217–218.

[B43] Ward E. DeSantis C. Robins A. Kohler B. Jemal A. (2014). Childhood and adolescent cancer Statistics, 2014. *CA Cancer J. Clin.* 64 83–103. 10.3322/caac.21219 24488779

[B44] Wilne S. Collier J. Kennedy C. Koller K. Grundny R. Walker D. (2007). Presentation of childhood CNS tumours: a systematic review and meta-analysis. *Lancet Oncol.* 8 685–695. 10.1016/S1470-2045(07)70207-3 17644483

[B45] Young K. Bowers A. Bradford N. (2021). Families’ experiences of child and adolescent brain tumors: a systematic review and synthesis of qualitative research. *Psychoonchology* 30 1643–1662. 10.1002/pon.5745 34124814

[B46] Zander M. Hutton A. King L. (2010). Coping and resilience factors in pediatric oncology nurses. *J. Pediatr. Oncol. Nurs.* 27 94–108. 10.1177/1043454209350154 20044588

[B47] Zonza M. (2012). Narrative based medicine and neonatology: an interpretative approach. *J. Pediatr. Neonatal Individualizaed Med.* 1 49–52.

